# Efficacy of MiraNet Combi, a novel alpha-cypermethrin, pyriproxyfen, and piperonyl butoxide long-lasting insecticidal net against pyrethroid-resistant *Anopheles funestu*s in western Kenya

**DOI:** 10.1371/journal.pone.0331995

**Published:** 2026-08-12

**Authors:** Mathew Kipsum, Silas Agumba, Vincent Moshi, Margaret Muchoki, Seline Omondi, Celestine Wekesa, Collins Ouma, Bernard Abong’o, Eric Ochomo

**Affiliations:** 1 Centre for Global Health, Kenya Medical Research Institute, Kisumu, Kenya; 2 Department of Biomedical Sciences and Technology, School of Public Health and Community Development, Maseno University, Maseno, Kenya; 3 Research World Limited Company, Kisumu, Kenya; 4 Vector Group, Liverpool School of Tropical Medicine, Liverpool, United Kingdom; 5 Centre for Infectious and Parasitic Disease Control Research, Kenya Medical Research Institute, Busia, Kenya; World Health Organization, Regional Office for South-East Asia, INDIA

## Abstract

**Background:**

The widespread use of pyrethroid-only long-lasting insecticidal nets (LLINs) has contributed substantially to malaria control; however, increasing pyrethroid resistance in malaria vectors threatens their continued effectiveness. To address this challenge, LLINs incorporating additional active ingredients have been developed. MiraNet® Combi is a novel LLIN incorporating alpha-cypermethrin, the insect growth regulator pyriproxyfen (PPF), and the synergist piperonyl butoxide (PBO).

**Methods:**

We evaluated the efficacy of MiraNet® Combi against pyrethroid-resistant *Anopheles funestus* in western Kenya using WHO cone bioassays, tunnel tests, experimental hut trials, and chemical analysis. Non-inferiority of MiraNet® Combi to Royal Guard® (alpha-cypermethrin + PPF) and superiority to Olyset® Plus (permethrin + PBO) and MiraNet® (alpha-cypermethrin-only) were assessed for mosquito mortality and blood-feeding inhibition following 20 standardized washes, in accordance with WHO guidelines.

**Results:**

In laboratory assays, MiraNet® Combi achieved high efficacy, inducing 94.5% mortality (95% CI: 91.2–97.8%) and 93.8% blood-feeding inhibition (95% CI: 90.5–97.1%) across all wash points against susceptible and resistant mosquito strains. In experimental hut trials, MiraNet® Combi produced the highest mortality, with 23% (95% CI: 21–25%) in unwashed and 25% (95% CI: 23–27%) in washed nets, significantly exceeding unwashed Olyset® Plus (19.9%, *p* = 0.0002) and MiraNet® (21.9%, *p* = 0.03). Blood-feeding inhibition was comparable across all ITNs (90.2–98.4%). Unwashed MiraNet® Combi reduced ovary development by 37.5% (95% CI: 32.1–42.9%), decreasing to 14.3% (95% CI: 10.2–18.4%) after washing. MiraNet® Combi was non-inferior to Royal Guard® for mosquito mortality but not for blood-feeding inhibition, superior to Olyset® Plus and MiraNet® for mosquito mortality, superior to MiraNet® for blood-feeding inhibition, and inferior to Olyset® Plus for blood-feeding inhibition.

**Conclusions:**

MiraNet® Combi provides improved personal protection and enhanced efficacy against pyrethroid-resistant *An. funestus* compared with existing LLINs. The combination of a pyrethroid, PBO, and PPF represents a promising strategy for resistance management and malaria vector control in areas with high pyrethroid resistance.

## Background

Malaria remains a major global public health challenge, with insecticide-treated nets (ITNs) serving as the primary preventive measure for vector control. [1]. However, the emergence and spread of insecticide resistance, particularly to pyrethroids, threatens the effectiveness of these interventions [2]. In response, significant collaborative efforts between chemical industries, academia, and product development partnerships to expand the vector control toolbox and manage pyrethroid resistance in malaria vectors. The World Health Organization (WHO) recommended pyrethroid-treated nets for malaria control until 2018. However, increasing pyrethroid resistance in malaria vectors has necessitated the development of nets incorporating additional active ingredients. [3,4]. Furthermore, novel net types are being developed to maintain effectiveness in resistance-prone areas, incorporating insecticide synergists, multiple insecticides, or combinations of insecticides and insect growth regulators [5].

Insecticide treated nets containing both pyrethroids and piperonyl butoxide (PBO; an insecticide synergist that primarily acts by inhibiting the action of oxidase enzymes in mosquito vectors) have been shown to be effective against pyrethroid-resistant *An. gambiae* sensu lato (s.l.) and *An. funestus* mosquitoes [1]. Recent large cluster randomized trials in Uganda [2] and Tanzania [3] showed that the pyrethroid-PBO nets Olyset^®^ Plus (Sumitomo Chemical, Tokyo, Japan) and PermaNet^®^ 3.0 (Vestergaard Frandsen, Lausanne, Switzerland) confer better protection against malaria than pyrethroid-only nets in areas where the main malaria vectors exhibit intermediate levels of resistance mediated by the monooxygenase-based pyrethroid resistance mechanism [4]. However, there is still uncertainty regarding the performance of pyrethroid-PBO ITNs against mosquito populations with extremely high levels of pyrethroid resistance or when there are multiple mechanisms of resistance, such as glutathione *S*-transferases (GST)-based metabolic resistance, driving the insecticide resistance phenotype [5]. This emphasizes the need for additional innovations to improve control and reduce malaria transmission.

The effectiveness of ITNs against insecticide-resistant *Anopheles* depends on the frequency and strength of the resistance [6–8]. To address this challenge, new active ingredients and combinations are being developed and tested. A promising approach is the incorporation of multiple compounds in ITNs, such as pyrethroids, the synergist piperonyl butoxide (PBO), and pyriproxyfen, an insect growth regulator. This combination aims to improve control of pyrethroid-resistant anopheline mosquitoes through multiple modes of action. Several ITNs with dual active ingredients have shown promising results in rigorous randomized controlled trials across various malaria-endemic settings. These include Interceptor® G2 (pyrethroid + chlorfenapyr), Royal Guard® (alpha-cypermethrin + pyriproxyfen), and Olyset® Duo (permethrin + pyriproxyfen) [4,9–11]. The epidemiological efficacy demonstrated by these next-generation ITNs highlights the potential of multi-ingredient approaches in combating insecticide resistance. Before novel ITNs can be recommended for public health use, they undergo a rigorous evaluation process by the WHO Pre-Qualification Team (PQT) [12]. This process ensures that new vector control tools meet the necessary safety and efficacy standards for widespread deployment.

While large-scale randomized controlled trials provide valuable epidemiological data on the effectiveness of next-generation ITNs, it is not feasible to conduct such trials in all epidemiological and ecological settings due to resource and time constraints. However, experimental hut trials offer a cost-effective alternative for assessing the entomological efficacy of these interventions [13]. Data from these experimental hut studies can be used in malaria transmission models to predict the effectiveness of novel ITNs in comparison to first-in-class products by conducting non-inferiority assessments [14]. MiraNet® Combi (A to Z Group Limited, Tanzania) is a novel triple-active ITN that combines alpha-cypermethrin, PBO, and pyriproxyfen (PPF). The addition of PPF, a juvenile hormone mimic, aims to reduce the fecundity and fertility of adult *Anopheles* mosquitoes that survive contact with the net [15]. This multi-pronged approach is designed to enhance vector control in areas with high frequency and intensity of insecticide resistance. Despite the availability of dual-active LLINs incorporating either PBO or pyriproxyfen, evidence on the added value of combining a pyrethroid, a synergist, and an insect growth regulator within a single net remains limited. MiraNet® Combi was developed to address this gap by targeting both metabolic resistance mechanisms and mosquito reproductive capacity, thereby offering a potentially more durable resistance-management tool.

The present study evaluated the non-inferiority of MiraNet® Combi against two established combination ITNs: Royal Guard® (alpha-cypermethrin + pyriproxyfen) and Olyset® Plus (permethrin + PBO). Additionally, we assessed the superiority of MiraNet® Combi over the standard pyrethroid-only MiraNet® ITN. This evaluation was conducted using experimental hut trials against wild, free-flying pyrethroid-resistant *An. funestus* in Siaya, western Kenya.

## Methods

### Study site and experimental huts

**Fig 1:**
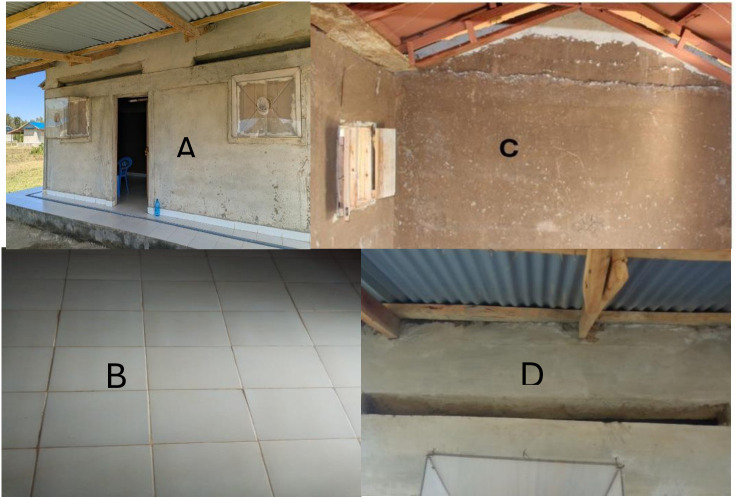
Experimental hut design: A front view of the hut fitted with window exit traps, B showing the tiled floor and the hut interior walls C showing the wood baffles and D showing eaves.

Experimental hut trials (EHTs) were conducted at the *Dala Suna* experimental hut site on the shores of Lake Kanyaboli (0° 02′ 08.5″ N, 34° 11′ 05.0″ E) in Alego Usonga sub-County, Siaya County, western Kenya. The huts are located close to the swamps that provide conducive breeding habitats for malaria vectors and are characterized by a high year-round abundance of *An. funestus* and seasonal peaks of *An. arabiensis*, with average household densities > 300 and > 20 per night, respectively at peak seasons [16]. The area experiences two rainy seasons, from March to May and October to November, with high malaria transmission throughout the year [17]. The experimental huts are designed to resemble a typical Kenyan household structure with mosquito exit/entry points (eaves, windows and doors) (Fig 1A). Mosquito exit traps were fitted to all four windows of the experimental huts, two windows on the front face and two on the back of the huts. The walls are made of blocks and lined with mud on the inside. The huts have corrugated iron roofs and a 10-cm eave gap. To prevent mosquitoes from exiting the huts, wood baffles are installed at the eave gaps, allowing easy entry for mosquitoes (Fig 1C). In addition, the floors are tiled white for easy collection of knocked-down and dead mosquitoes (Fig 1B). The huts are elevated above the ground on a concrete base surrounded by a water-filled moat to keep ants away [18].

### Insecticides susceptibility

Mosquito larvae and adults were collected from the study area between May and July 2024. *An. gambiae* s.l. larvae were collected from natural breeding sites, while adult blood-fed *An. funestus* were collected indoors from surrounding houses after obtaining informed consent. Mosquitoes were reared at the KEMRI-CGHR insectary under standard conditions (27 ± 2°C, 80 ± 10% RH, 12:12 light: dark cycle). The F1 generation of *An. funestus* and adult *An. gambiae* s.l. were used for insecticide resistance testing. The insecticide-susceptible Kisumu strain of *An. gambiae* served as a control. Insecticide susceptibility was assessed using WHO tube and bottle assays. Three- to five-day-old mosquitoes were exposed to diagnostic doses of alpha-cypermethrin (0.05%), permethrin (0.75%), and deltamethrin (0.05%) for 1 hour. Knockdown was recorded every 10 minutes during exposure, and mortality was assessed 24 hours post-exposure. Resistance intensity was evaluated using 5X and 10X diagnostic concentrations. Synergist assays were conducted by pre-exposing mosquitoes to 4% PBO for 1 hour before insecticide exposure. Bottle bioassays were conducted using chlorfenapyr (100 µg/bottle) and clothianidin (4 µg/bottle) following WHO protocol [21]. Mosquitoes were exposed for 60 minutes, with knockdown recorded every 10 minutes. Post-exposure mortality was monitored at 24, 48, and 72 hours for chlorfenapyr, and at 24 hours for clothianidin.

### Description of treatments used in the EHTs

1. MiraNet® Combi (A to Z Group Limited, Tanzania): HDPE monofilament net incorporating alpha-cypermethrin (6.0 g/kg), pyriproxyfen (20 g/kg), and PBO (10 g/kg).
2. Olyset® Plus (Sumitomo Chemical Company Limited, Japan): Monofilament HDPE LLIN with permethrin (20 g/kg) and PBO (10 g/kg).
3. Royal Guard® (Disease Control Technologies, USA): HDPE/LLDPE fabric monofilament net treated with alpha-cypermethrin and pyriproxyfen (both at 5.0 g/kg).
4. MiraNet® (A to Z Group Limited, Tanzania): Standard pyrethroid LLIN treated with alpha-cypermethrin (4.5 g/kg).

### Baseline quality check and net washing

Forty-five nets (15 each of MiraNet® Combi, MiraNet®, and Olyset® Plus) were randomly selected for baseline quality assessment, for the Royal Guard® this was not done since the number supplied was fixed. Three 25 cm x 25 cm samples from each net were tested using cone assays and chemical analysis following WHO guidelines.

Nine nets of each type were washed 20 times according to WHO protocols [22]. Nets were washed individually in 10 L of clean water with 2 g/L of soap, agitated for 10 minutes, and rinsed twice. Nets were dried under shade and stored at ambient temperature between washes. All nets used in the experimental hut trials were deliberately holed with six 4 x 4 cm holes to simulate wear and tear [23].

### Laboratory assays

#### Cone test.

Cone bioassays were performed on 25 cm x 25 cm net pieces before and after field trials for all wash points. Five non-blood-fed female mosquitoes were exposed to each of four cones attached to the net piece for 3 minutes [24]. Both susceptible *An. gambiae* (Kisumu strain) and F1 *An. funestus* were tested. Knockdown was recorded at 60 minutes post-exposure, and mortality was assessed at 24, 36, and 72 hours.

#### Tunnel test.

Tunnel tests were conducted to assess host-seeking behavior, mortality, and blood-feeding success of pyrethroid-resistant *An. funestus* F1 mosquitoes. Tests were performed using the same net pieces as in the cone assays. The tunnel apparatus consisted of a square glass chamber divided into two sections by a frame fitted with a net sample. A rabbit was used as bait in the shorter section, while 100 5–8-day old mosquitoes were released in the longer section at 1800 hours and left until 0700 hours the next day. Mortality and blood-feeding success were recorded, with delayed mortality assessed up to 72 hours post-exposure.

### Experimental hut trial procedure

Experimental hut trials were conducted to compare the entomological efficacy of MiraNet^®^ Combi, Royal Guard^®^, Olyset® Plus and MiraNet^®^ ITNs, tested unwashed and after 20 standardized washes, against free flying pyrethroid-resistant *An. funestus*. Each ITN type included nine unwashed and nine washed replicates. The untreated control comprised nine nets, giving a total of 81 nets assessed.

Nine consented volunteers slept in the huts from 20:30–06:30 daily throughout the trial period. To control variation in individual attractiveness to mosquitoes, volunteers rotated daily between the huts using a simple 9*9 Latin Square Design. Treatments were also rotated between huts after each 9-night collection period to minimize positional effects. Nets were installed following standard hut procedures, with the roof panel edges secured to nails fixed at the upper corners of the hut walls. Mosquito collections were performed for 9 days in each collection round; on the 10th day, the huts were cleaned and aired to prevent contamination and carry-over effects before the next rotation cycle.

The trial evaluated the following treatment arms, each with nine replicates: untreated control (washed 20 times), MiraNet® Combi (unwashed and washed), Royal Guard® (unwashed and washed), Olyset® Plus (unwashed and washed), and MiraNet® (unwashed and washed).

#### Mosquito collections and processing.

Nine consented volunteers slept in experimental huts from 20:30–06:30 during each trial to attract wild, free-flying mosquitoes. All volunteers were provided with weekly prophylaxis (Mefloquine) and supervisors recorded side effects throughout the study period. Mosquito collections were performed daily from 06:30–08:00 using mouth aspirators. Sleepers collected both dead and alive mosquitoes from inside the huts, including the walls, roof, inside and under the bed net, and from the window exit traps using mouth aspiration. Collected mosquitoes were transferred into clean, netted paper cups and provided with a 10% sugar solution. Samples were placed in cooler boxes and transported to the field insectary laboratory.

In the laboratory, the mosquitoes were sorted by physiological status (alive or dead, blood-fed or unfed, gravid or half-gravid) and identified morphologically to species using standard taxonomical keys [19]. All the live mosquitoes were observed for knockdown one-hour post-collection, and mortality was recorded at 24-hour interval for up to 72 hours.

To evaluate the impact of MiraNet^®^ Combi and Royal Guard^®^ ITNs on mosquito reproduction, subsamples of surviving blood-fed mosquitoes were dissected post-survival (72hr) to observe ovarian development. Fertility was scored according to Christophers’ stages of egg development [20],with females classified as fertile if oocytes reached stage V and infertile if development remained at stages I–IV.

### Experimental hut trial outcome measures

The primary outcome measures used to express the efficacy of the experimental hut treatments against pyrethroid-resistant *An. funestus* and compare the impact of the MiraNet^®^ Combi ITNs to the pyrethroid-only ITNs MiraNet^®^ were:

i. **Mortality (%)**: the proportion of dead mosquitoes 72 h after collection
ii. **Blood-feeding (%)**: the proportion of blood-fed mosquitoes.
iii. **Blood-feeding inhibition (%)**: the reduction in the proportion of blood-fed mosquitoes in the treated hut relative to the untreated control hut. Calculated as follows:

$$\begin{array}{c} Blood\;feeding\;inhibition\;(\%)=\frac{\text{1}\text{0}\text{0}\;(Bfu-Bft)\;}{Bfu} \end{array}$$

Where *Bfu* is the proportion of blood-fed mosquitoes in the untreated control hut and *Bft* is the proportion of blood-fed mosquitoes in the treated hut.

iv. **Fertility (%)**: the proportion of dissected mosquitoes scored fertile.

The secondary outcome measures were used to express the efficacy of the experimental hut treatments against pyrethroid-resistant *An. funestus* were:

i. **Entry (*n*)**: the number of mosquitoes collected inside the hut.
ii. **Deterrence (%)**: the reduction in entry in the treated hut relative to the untreated control hut. Calculated as follows:

$$\begin{array}{c} Deterrence\;(\%)=\;\frac{\text{1}\text{0}\text{0}\;(Tu-Tt)}{Tu} \end{array}$$

Where *Tu* is the number of mosquitoes entering the untreated control hut and *Tt* is the number of mosquitoes entering the treated hut.

iii. **Exophily (%)**: exiting rates due to the potential irritant effect of treatments expressed as the proportion of mosquitoes collected in the window exit traps.
iv. **Reduction in fertility (%):** the proportion of dissected mosquitoes scored as fertile for a given treatment compared to the control. Calculated as follows:

$$\begin{array}{c} \text{Reduction in fertility }(\%)=\;\frac{\text{1}\text{0}\text{0}(Fu-Ft)}{Fu} \end{array}$$

Where *Fu* is the proportion of fertile mosquitoes in the untreated control hut and *Ft* is the proportion of fertile mosquitoes in the treated hut.

v. **Personal protection (%):** Reduction in the *actual number* of mosquitoes that blood-feed on a person. Calculated as:

$$\begin{array}{c} \text{Personal protection}=\;\frac{\text{1}\text{0}\text{0}(cu-ct)}{cu} \end{array}$$

Where Cu is the number of blood-fed mosquitoes in the untreated control hut and Ct is the number of blood fed in the treatment hut.

vi. **Overall killing effect:** is the proportionate increase in mosquito mortality attributable to an intervention compared to the untreated control. Calculated as

$$\begin{array}{c} Overall\;killing\;effect\;(\%)=\;\frac{\text{1}\text{0}\text{0}\;(Kt-Ku)}{Tu} \end{array}$$

Where:

Kt = number of mosquitoes **killed in the treatment hut**

Ku = number of mosquitoes **killed in the control hut**

Tu = total number of mosquitoes **collected in the control hut**

### Chemical assays

The net pieces for chemical analysis were cut from randomly selected ITNs from all the wash points in every arm, before and after the hut trials, and five pieces were obtained from each net apart from MiraNet Combi, from which 3 pieces were obtained from the top and 1 from each side (7 pieces) following WHO guidelines on net cutting. The cut net pieces were shipped wrapped in aluminium foil to the Department of Analytical Chemistry, International Institute of Biotechnology and Toxicology (IIBAT), Padappai – 601 301, Kancheepuram District, Tamil Nadu, India to determine the wash retention of active ingredients in the net pieces using analytical methods validated and published by the Collaborative International Pesticides Analytical Council (CIPAC).

The active content of Alpha-cypermethrin in MiraNet Combi, Royal Guard and MiraNet ITNs was quantified by capillary Gas Chromatography using Flame Ionization Detector (GC-FID) and dioctyl phthalate as internal standard, after extraction by refluxing with xylene and 10% citric acid solution for 30 minutes. Citric acid was added to avoid the epimerization of Alpha-cypermethrin. Alpha-cypermethrin content was analyzed following CIPAC volume M; 454/LN/M/3.2.

The active content of Piperonyl butoxide in MiraNet Combi and Olyset® Plus was quantified by capillary Gas Chromatography using Flame Ionization Detector (GC-FID) and octadecane as an internal standard, after extraction by refluxing with xylene for 30 minutes. Piperonyl butoxide content was analyzed following CIPAC volume N; CIPAC/4941, extension of 33/LN/(M)/3.

The active content of Pyriproxyfen in MiraNet Combi Roof Panel and Royal Guard was quantified using HPLC and dicyclohexyl phthalate as internal standard. After being evaporated, added acetonitrile dissolved completely. Pyriproxyfen content was analyzed following CIPAC Volume – N, CIPAC/4887, extension of 715/TC/M/3 and 715/LN/M/3.

The active content of Permethrin in Olyset® Plus was quantified by capillary Gas Chromatography using Flame Ionization Detector (GC-FID) and dicyclohexyl phthalate as internal standard, after evaporating filter the solution was injected for analysis Permethrin content was analyzed following (CIPAC/4841).

### Data analysis

The difference in proportional outcomes (mortality, blood feeding and exophily) between treatments and control at all wash points were analysed using binomial generalized linear mixed-effects models with hut ID, sleeper ID and collection week as random effects. In contrast, differences in numerical outcomes (entry) were analysed using a negative binomial regression model. Tests of non-inferiority between MiraNet^®^ Combi and Royal Guard^®,^ as well as Olyset® Plus for both mortality and blood feeding, were performed according to the WHO protocol [21]. A candidate product is considered non-inferior to the active comparator product if: (a) the lower 95% confidence interval of the odds ratio describing the difference in mortality between the candidate and comparator product is > 0.7 and/or (b) the upper 95% confidence interval of the odds ratio describing the difference in blood feeding between the candidate and comparator product is < 1.43. The superiority between MiraNet^®^ Combi and MiraNet^®^ was also assessed based on whether mortality rates were higher and blood feeding rates lower at a 5% significance level (i.e., p < 0.05). All analyses were done using R Statistical Software (v4.2.2; R Core Team 2021).

### Ethical Considerations and Compliance with Good Laboratory Practice (GLP)

Recruitment start day 04/01/2024 and ended in 05/11/2024

This study was approved by KEMRI Scientific and Ethical Review Unit (Ref: SERU 4536). All adult participants (aged 18–45 years) provided **written informed consent** prior to participation. Consent forms were provided in both English and Dholuo, and participants signed the form before enrolment. For those unable to write, a thumbprint was obtained in the presence of an impartial witness, who also signed the form. The study did not involve minors; therefore, parental or guardian consent was not required. No waivers of consent were requested or granted by the ethics committees. Those who consented were provided with weekly malaria prophylaxis (Mefloquine). The study site is accredited by the Kenya Pest Control Products Board (PCPB) for national evaluation of vector control products. All procedures were conducted in accordance with WHO guidelines for non-inferiority trials of LLINs [27] and in compliance with Good Laboratory Practice (GLP) requirements.

## Results

### Insecticide susceptibility assays

A total of 3,000 female mosquitoes were tested for insecticide susceptibility. No mortality was observed in control groups, eliminating the need for Abbott’s formula correction. Pyrethroid resistance was detected in both *An. gambiae s.l.* and *An. funestus* populations (Table 1). *An. gambiae s.l.* showed 45% mortality to the diagnostic dose of deltamethrin (1X), increasing to 84% at 5X dose. Similar patterns were observed for permethrin and alpha-cypermethrin. *An. funestus* exhibited 72% mortality to 1X deltamethrin, rising to 77% at 5X dose. *An. funestus* did not exhibit 100% mortality even at 10X the diagnostic doses. Pre-exposure to PBO fully restored susceptibility to deltamethrin and partially restored susceptibility to permethrin and alpha-cypermethrin in *An. gambiae s.l.* For *An. funestus*, PBO only partially restored susceptibility to all tested pyrethroids. Non-pyrethroid insecticides (pirimiphos-methyl, clothianidin, and chlorfenapyr) induced 100% mortality at diagnostic doses in both species.

**Table 1:** Insecticide resistance profile of Anopheles malaria vector population from the Lake Kanyaboli experimental hut site.

Assays	Insecticide	Concentration	*An. gambiae* s.l.		*An. funestus*	
			*Sample size*	*% mortality*	*Sample size*	*% mortality*
WHO tube	Alpha-cypermethrin	0.05%	100	82	100	45
		0.25%	100	88	100	60
		0.50%	100	93	100	94
	PBO + Alpha-cypermethrin	0.05%	100	95	100	97
	Deltamethrin	0.05%	100	45	100	72
		0.25%	100	84	100	77
		0.50%	100	100	100	92
	PBO +Deltamethrin	0.05%	100	100	100	97
	Permethrin	0.75%	100	82	100	64
		3.75%	100	98	100	86
		7.50%	100	100	100	94
	PBO + Permethrin	0.75%	100	99	100	95
	Pirimiphos methyl	0.25%	100	100	100	100
WHO bottle	Clothianidin	4 µg/ml	100	100	100	100
	Chlorfenapyr	100 µg/ml	100	100	100	100

### Baseline variability check

**Fig 2:**
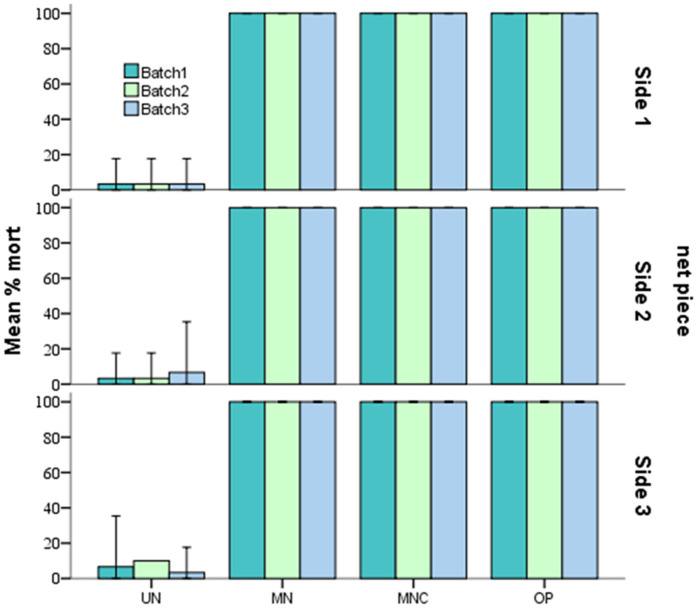
Mortality of An. gambiae, Kisumu strain against three batches of ITNs before the hut evaluation. UN = untreated net; MN = MiraNet®; MNC = MiraNet® Combi; OP = Olyset® Plus; RG = Royal Guard.

All three batches of the assessed LLINs induced 100% mortality within 24 hours when exposed to the susceptible *An. gambiae* Kisumu strain, indicating no significant variation between batches (Fig 2).

### Supplementary assays

#### Tunnel assay.

Before the hut trial, MiraNet® Combi, Royal Guard®, and Olyset® Plus induced >96% mortality at all wash points against pyrethroid-resistant *An. funestus*. MiraNet® showed a slight decrease in mortality from 97% (unwashed) to 93.5% (20 washes). Post-hut trial, MiraNet® Combi and Olyset® Plus maintained >96% mortality, while Royal Guard® efficacy decreased to 93–95% (Table 2). All tested LLINs induced 100% mortality in susceptible *An. gambiae* Kisumu strain.

**Table 2:** Proportion of dead An. funestus when exposed to ITNs in tunnel assays.

ITNs	Before the hut trial		After the hut trial	
	Unwashed % (N)	Washed 20X % (N)	Unwashed % (N)	Washed 20X % (N)
MiraNet^®^	97 (194)	93.5 (187)	93 (187)	95 (190)
MiraNet^®^ Combi	98 (196)	97.5 (195)	97 (194)	96.5 (193)
Olyset® Plus	97 (194)	99.5 (199)	97.5 (195)	96 (192)
Royal Guard^®^	96.5 (193)	96.5 (193)	93(192)	95 (193)

**Fig 3:**
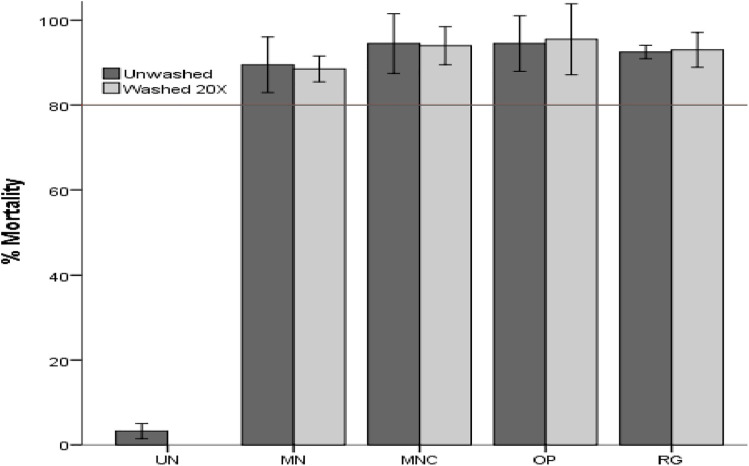
Mortality of pyrethroid-resistant An. funestus, from the experimental hut site, when exposed to wash-resistance tunnel tests. Error bars represent 95% confidence intervals. The red lines indicate the WHO cut-off criteria for efficacy in tunnels. UN = untreated net; MN = MiraNet®; MNC = MiraNet® Combi; OP = Olyset® Plus; RG = Royal Guard.

**Fig 4:**
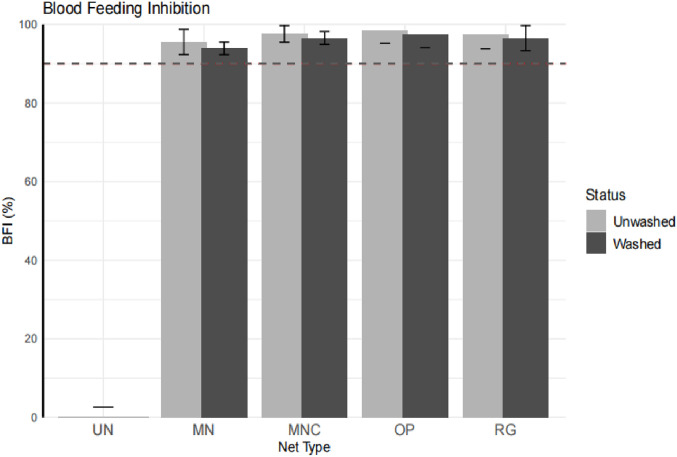
Blood-feeding inhibition of pyrethroid-resistant An. funestus, from the experimental hut site in Siaya, when subjected to wash-resistance tunnel tests. Error bars represent 95% confidence intervals. The red lines indicate the WHO cut-off criteria for efficacy in tunnels. UN = untreated net; MN = MiraNet®; MNC = MiraNet® Combi; OP = Olyset® Plus; RG = Royal Guard.

MiraNet^®^ Combi demonstrated high efficacy in wash resistance tunnel tests, with ≥95% mortality (Fig 3) and ≥94% blood-feeding inhibition against resistant strains across all wash points (Fig 4).

#### Cone assay.

**Fig 5:**
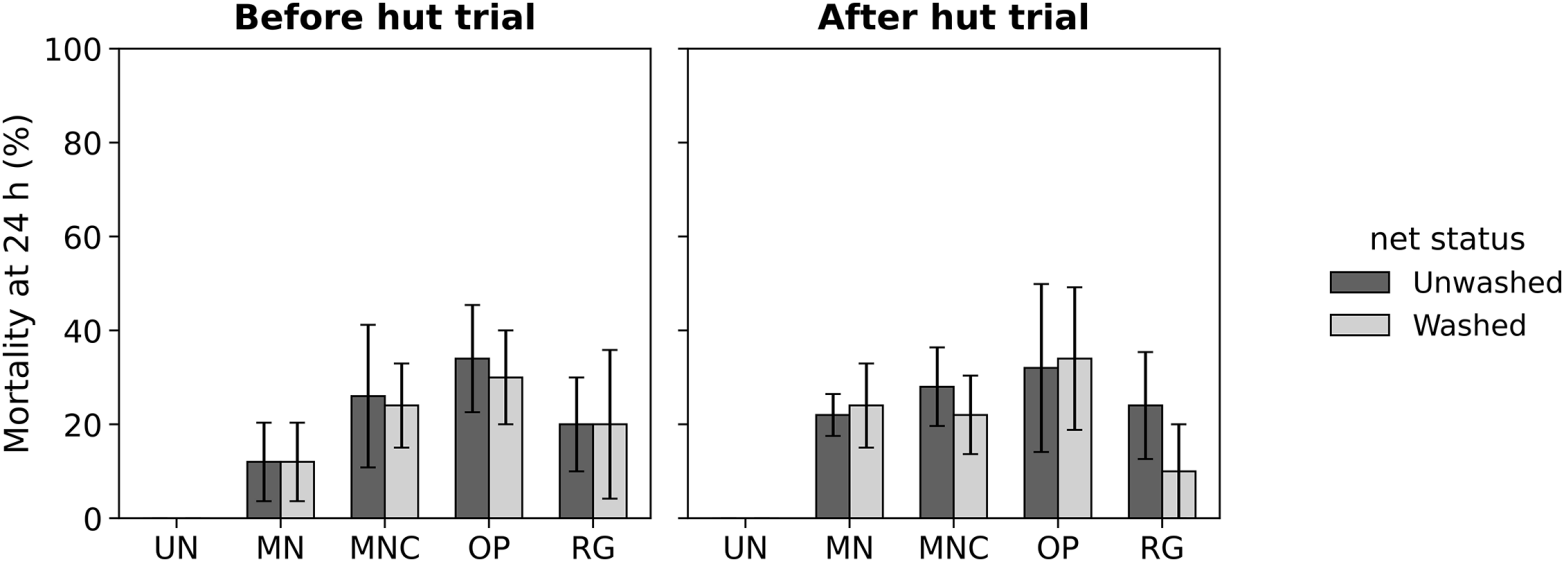
Mortality of pyrethroid-resistant An. funestus, from the experimental hut site, Siaya, when exposed in the cone assay. UN = untreated net; MN = MiraNet®; MNC = MiraNet® Combi; OP = Olyset® Plus; RG = Royal Guard.

**Fig 6:**
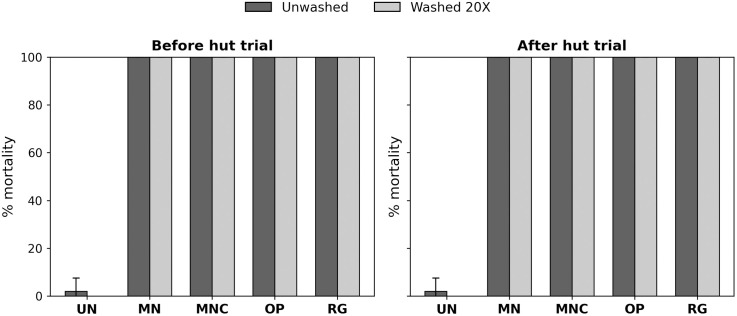
Mortality of susceptible An. gambiae, Kisumu strain, laboratory colony when exposed cone assay for three minutes. Error bars represent 95% confidence intervals. UN = untreated net; MN = MiraNet®; MNC = MiraNet® Combi; OP = Olyset® Plus; RG = Royal Guard.

Cone bioassays with *An. funestus* resulted in relatively low mortality rates. Before hut trials, MiraNet® Combi and Olyset® Plus induced 44–48% mortality, Royal Guard® 42%, and MiraNet® 34–36%. Post-trial mortality rates decreased slightly for all LLINs (Fig 5). There was 100% mortality of An. gambiae Kisumu strain laboratory colony when exposed for three minutes in the cone assay (Fig 6).

### Chemical analysis

#### Quantification of active content.

The initial active ingredient (AI) concentrations of all unwashed nets were within manufacturer specifications (Table 3). After 20 washes, retention rates varied considerably between active ingredients and net types: Alpha-cypermethrin showed consistent retention across products: MiraNet Combi: 79.3%, Royal Guard: 81.2%, and MiraNet: 74.8%. PBO retention differed between products: MiraNet Combi: 74.1% and Olyset® Plus: 66.9%. Pyriproxyfen showed lowest retention: MiraNet Combi: 56.8% and Royal Guard: 61.3%. Permethrin in Olyset® Plus showed 74.0% retention. Panel location (top vs side) had minimal impact on retention for most AIs (<5% difference), except for pyriproxyfen in Royal Guard which showed lower retention in top panels (56.8%) compared to side panels (59.2%). Statistical comparison showed no significant differences in alpha-cypermethrin retention between net types (p > 0.05). However, PBO retention was significantly higher in MiraNet Combi compared to Olyset® Plus (p < 0.01). All nets met WHO criteria for minimum active ingredient content after 20 washes (>75% for pyrethroids).

**Table 3:** The content of active ingredients contained in unwashed and washed net pieces before and after hut trial in the experimental hut trial in Siaya, western Kenya.

ITNs	Active Ingredient (AI)	Panel	Quantity of AI/Wash status/Panel		AI Retention/Panel (%)	AI Retention/ITN (%)
			Unwashed (g/kg)	Washed 20X (g/kg)		
MiraNet	Alpha-cypermethrin	Side	4.76	3.	76.68	77.04
		Top	4.78	3.7	77.41	
MiraNet Combi	Alpha-cypermethrin	Side	5.61	4.		82.71
	Pyriproxyfen (PPF)	Top	20.41	11.7	57.32	57.32
	Piperonyl butoxide (PBO)	Side	9.99	7.60	76.09	76.00
		Top	9.96	7.56	75.90	
Olyset® Plus	Permethrin	Side	19.51	15.68	80.37	79.81
		Top	19.71	15.62	79.25	
	Piperonyl butoxide	Side	8.37	5.87	70.13	70.87
		Top	8.31	5.95	71.60	
Royal Guard	Alpha-cypermethrin	Side	4.89	3.93	80.37	80.91
		Top	4.96	4.04	81.45	
	Pyriproxyfen (PPF)	Side	5.12	3.03	59.18	58.01
		Top	5.12	2.91	56.84	

### Experimental hut trials

#### Mosquito entry and exiting rates:.

**Fig 7:**
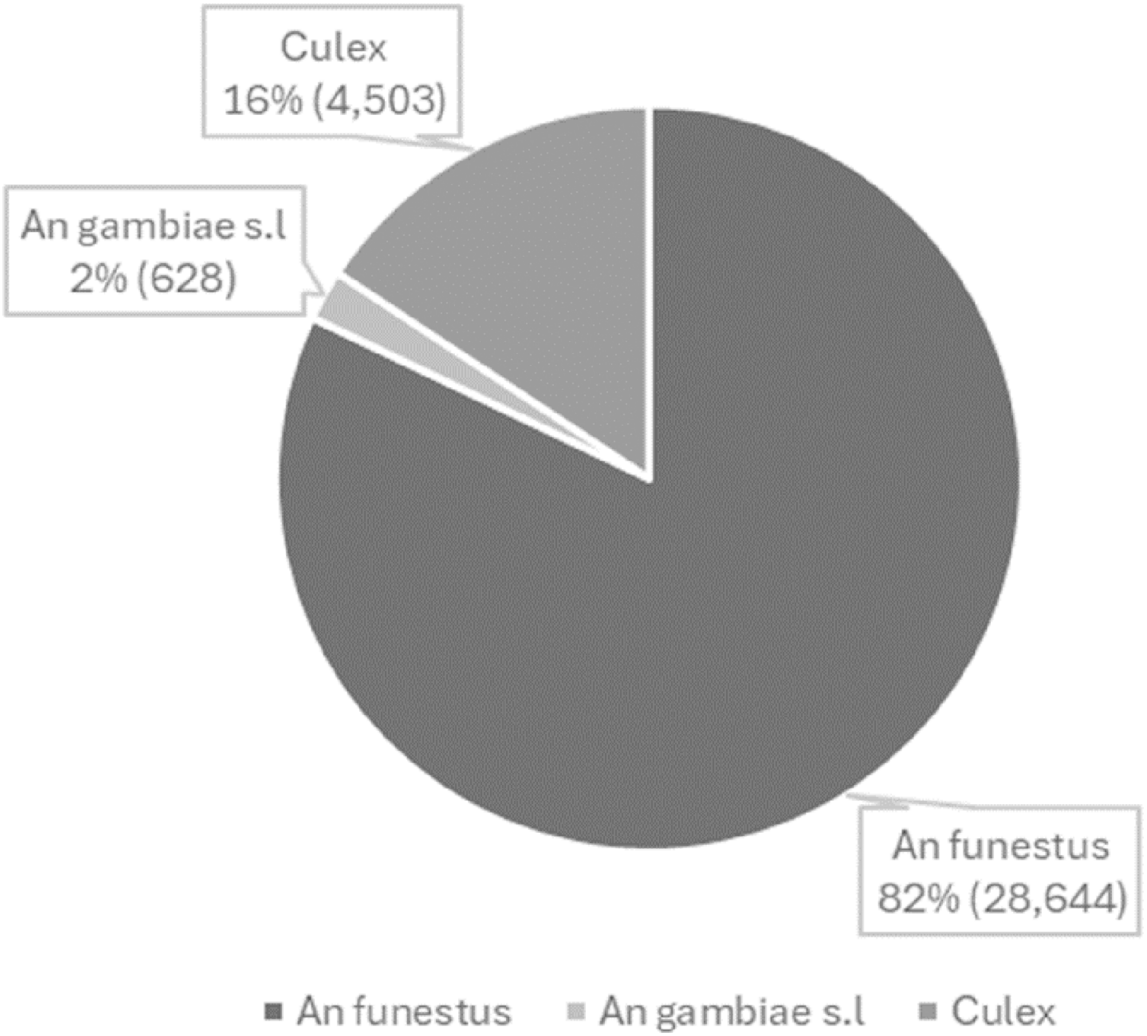
Proportion of mosquito population from EHTs site in Lake Kanyaboli, Siaya, western Kenya.

*An. funestus* was the dominant species (82%), followed by Culex (16%) and *An. gambiae s.l.* (2%) (Fig 7). Due to insufficient numbers of *An. gambiae s.l,* downstream analyses focused on *An. funestus*. A total of 15394 female *An. funestus* wild free-flying mosquitoes were collected in experimental huts during the trial (Table 4). The average nightly mosquito collection in control huts was 43. Mosquito entry decreased with washed nets across all ITNs, though these were not statistically significant. Exiting rates were significantly higher for all ITNs compared to the untreated net (5%). Among the ITNs, unwashed Olyset® Plus induced the highest exiting rate. Exiting decreased with washing for all ITNs except for Royal Guard®

**Table 4:** Rates of deterrence and exophily of mosquitoes in the different experimental arms.

Net type	Net status	Total females caught*	%Deterrence	Total exiting	% Exophily*	95% CIs
**Untreated net**	**–**	3500^b^	–	166	5^c^	4-6
**MiraNet** ^**®**^	**Unwashed**	1477^a^	58	457	31^a^	29-33
	**Washed 20x**	1423^a^	59	432	30^a^	28-33
**MiraNet**^**®**^ **Combi**	**Unwashed**	1545^a^	56	443	29^a^	26-31
	**Washed 20x**	1403^a^	60	290	21^b^	19-23
**Olyset® Plus**	**Unwashed**	1359^a^	61	601	44^c^	42-47
	**Washed 20x**	1293^a^	63	397	31^a^	28-33
**Royal Guard**	**Unwashed**	1891^b^	46	562	30^a^	28-32
	**Washed 20x**	1503^b^	57	496	33^a^	31-35

*Values in the same column bearing the same letter do not differ significantly at the 5% level

### Blood-feeding inhibition

**Fig 8:**
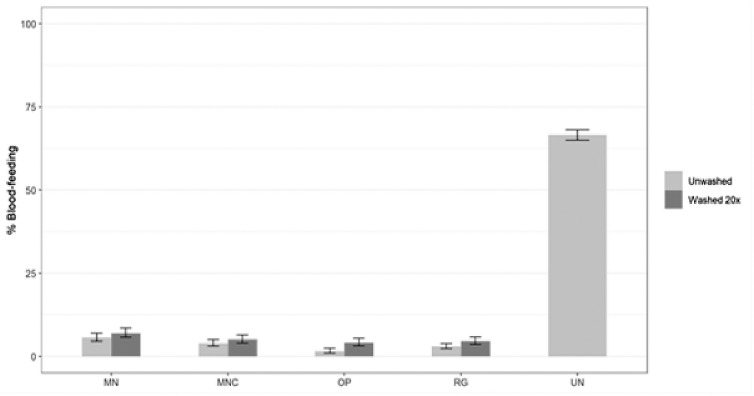
Blood feeding rates under the different types of nets. *UN = untreated net; MN = MiraNet®; MNC = MiraNet® Combi; OP = Olyset® Plus; RG = Royal Guard*.

Mosquito blood-feeding with the untreated net was at 67%. Relative to the control, blood-feeding rates were significantly lower for all treatment arms (Fig 8). Olyset® Plus had the lowest blood feeding rate amongst all treated ITN for both unwashed and washed categories. With all ITN types, blood-feeding rates increased after 20 washes. Blood-feeding inhibition was highest with Olyset® Plus (98% before washing and 94% after 20 washes) and lowest with MiraNet^®^ (92% before washing and 90% after 20 washes). Overall, all ITNs had very high and similar rates of blood-feeding inhibition. This was also the case for personal protection (Table 5).

**Table 5:** Blood-feeding inhibition rates of new generation ITNs against *An. funestus* in experimental huts at Lake Kanyaboli, western Kenya. Values in the same column bearing the same letter do not differ significantly at the 5% level.

Net type	Net status	Total females caught*	Total blood-fed	% Blood feeding*	95% CIs	% Blood feeding inhibition	% Personal protection
**Untreated net**	**–**	3500^c^	2329	67^e^	65-68	–	–
**MiraNet®®**	**Unwashed**	1477^ab^	83	6^ab^	5-7	92	96
	**Washed 20x**	1423^a^	99	7^c^	6-8	90	96
**MiraNet® Combi®**	**Unwashed**	1545^ab^	60	4^ab^	3-5	94	97
	**Washed 20x**	1403^a^	71	5^a^	4-6	92	97
**Olyset® Plus**	**Unwashed**	1359^a^	21	2^d^	1-2	98	99
	**Washed 20x**	1293^a^	53	4^ab^	3-5	94	98
**Royal Guard®**	**Unwashed**	1891^b^	56	3^b^	2-4	96	98
	**Washed 20x**	1503^ab^	69	5^ab^	4-6	93	97

### Reproductive effects

Mosquitoes from untreated huts had a 100% success rate in ovary development with all of them achieving Christopher’s stage V. However, fed mosquitoes that encountered PPF-treated nets were very low in number thus very few were available for dissections so these results must be interpreted with caution as the sample sizes were very low (Table 6). The unwashed MiraNet^®^ Combi net was found to inhibit ovary development by 37.5%, while the washed MiraNet^®^ Combi net had the lowest inhibition of 14.3%. In contrast, mosquitoes collected from huts with unwashed Royal Guard^®^ nets had a success rate of 100% in ovary development, while washed Royal guard^®^ had ovary development inhibition of 12.5%.

**Table 6:** Reproductive effects of pyriproxyfen-treated ITNs against pyrethroids-resistant *An. funestus* from experimental huts.

LLIN code	Wash point	Development stage	Number of mosquitoes dissected	% ovary development stage	% ovary development success rate
**MiraNet®**^**®**^ **Combi**	**Unwashed**	V	5	62.5	62.5
		I &IV	3	37.5	
	**Washed**		6	85.7	85.7
		I & IV	1	14.3	
**Royal Guard**	**Unwashed**	V	5	83.3	100
		I & IV	1	16.7	
	**Washed**	V	14	87.5	87.4
		I & IV	2	12.5	
**Control**	**Unwashed**	V	81	100.0	100

### Mortality

Control mortality at 72 hours was 6%. All ITNs induced significantly higher mortality compared to the control. MiraNet® Combi demonstrated the highest mortality before and after washing. In the unwashed category, the difference was only significant compared to unwashed Olyset® Plus. In the washed group, differences relative to MiraNet® and Olyset® Plus were statistically significant (p < 0.05). All the mortality was 25% and below indicative of the high level of insecticide resistance in this vector population (Table 7).

**Table 7:** Mortality at 72 hours induced by the different treatments. Values in the same column bearing the same letter do not differ significantly at the 5% level.

Net type	Net status	Total females caught*	Total 72h mortality	% 72h mortality*	95% CIs	% Overall killing effect
**Untreated net**	**–**	3500^c^	212	6^c^	5-7	–
**MiraNet** ^**®**^	**Unwashed**	1477^ab^	324	22^a^	20-24	3
	**Washed 20X**	1423^a^	305	21^a^	19-24	3
**MiraNet**^**®**^ **Combi**	**Unwashed**	1545^ab^	357	23^a^	21-25	4
	**Washed 20X**	1403^a^	348	25^c^	23-27	4
**Olyset® Plus**	**Unwashed**	1359^a^	271	20^b^	18-22	2
	**Washed 20X**	1293^a^	251	19^ab^	17-22	1
**Royal Guard** ^**®**^	**Unwashed**	1891^b^	406	21^ab^	20-23	6
	**Washed 20X**	1503^ab^	335	22^abc^	20-24	4

### Non-inferiority assessment

MiraNet Combi® was found to be superior to both Olyset® Plus and MiraNet® in terms of its ability to kill mosquitoes and to MiraNet® in terms of preventing vector blood feeding. It is Inferior to Olyset® Plus when it comes to preventing vector blood feeding. MiraNet Combi® was found to be non-inferior to Royal Guard® in terms of its ability to kill mosquitoes and not non-inferior to Royal Guard® in preventing vector blood feeding (Table 8).

**Table 8:** Assessment of superiority of Miranet Combi® to Olyset® Plusand Miranet® and non-inferiority to Royal Guard®.

		Superiority assessment		Superiority assessment		Non-inferiority assessment	
		Olyset® Plus	MiraNet Combi®	MiraNet®	MiraNet Combi®	Royal Guard®	MiraNet Combi®
Total	Total collected	2652	2948	2900	2948	3394	2948
Mortality	Total dead	522	705	629	705	741	705
	Mortality (%)	20	24	22	24	22	24
	Odds ratio	–	1.277	–	1.149	–	1.154
	Std. error (on log odds scale)	–	0.06664	–	0.064295	–	0.062039
	P-value	–	0.000244	–	0.0303	–	0.0212
	95% CIs	–	1.121 - 1.456	–	1.013 - 1.304	–	1.021 - 1.303
	WHO efficacy criteria	–	Significantly higher (p < 0.05)	–	Significantly higher (p < 0.05)	–	Lower 95% CI > 0.7
	Conclusion	–	**Superior**	–	**Superior**	–	**Non inferior**
Blood feeding	Total blood-fed	74	131	182	131	125	131
	Blood-feeding (%)	3	4	6	4	4	4
	Odds ratio	–	1.683	–	0.758	–	1.286
	Std. error (on log odds scale)	–	0.1531	–	0.12446	–	0.1352
	P-value	–	0.000677	–	0.026	–	0.0630
	95% CIs	–	1.249 - 2.282	–	0.593 - 0.967	–	0.986 - 1.677
	WHO efficacy criteria	–	Significantly lower (p < 0.05)	–	Significantly lower (p < 0.05)	–	Upper 95% CI <1.43
	Conclusion	–	**Inferior**	–	**Superior**	–	**Not Non inferior**

## Discussion

This study provides comprehensive evidence on the efficacy and wash resistance MiraNet® Combi, evaluating both chemical retention and bioefficacy against pyrethroid-resistant *An. funestus* in western Kenya. Our findings demonstrate three key outcomes that advance understanding of combination net performance. First, chemical analysis revealed differential retention of active ingredients after standardized washing. Alpha-cypermethrin showed highest retention (79.3%), followed by PBO (74.1%), while pyriproxyfen showed lowest retention (56.8%). These findings align with previous studies of combination nets [[5,22]] and highlight the technical challenge of maintaining multiple active ingredients through repeated washing. Second, despite variable chemical retention, MiraNet® Combi demonstrated non-inferiority to existing combination nets and superiority over standard nets in key efficacy measures. The mortality rates (23–24%) were significantly higher than standard nets (p < 0.05) and comparable to other combination products ([3]). The relatively low mortality observed across treatments reflects the high level of pyrethroid resistance in the local *An. funestus* population; however, MiraNet® Combi provided improved personal protection through enhanced blood-feeding inhibition. [23]. Third, the high blood-feeding inhibition (90–98%) maintained after washing suggests that personal protection remains robust even as insecticidal efficacy declines. This finding supports previous evidence that ITNs continue to provide valuable protection in settings with resistant mosquitoes through multiple mechanisms [24] and forms a robust argument against using insecticide free nets.

Although MiraNet® Combi contains a higher alpha-cypermethrin loading than some comparator nets, its improved performance cannot be attributed to pyrethroid dosage alone. The inclusion of PBO enhances pyrethroid bioavailability by inhibiting metabolic resistance mechanisms, while pyriproxyfen disrupts mosquito reproductive development. The combined action of these components is therefore likely responsible for the observed improvements in efficacy.

The discrepancy between high mortality rates in tunnel tests (87.5–99.5%) and lower rates in experimental hut trials reflects important real-world considerations. This exposure-dependent efficacy, documented in previous studies [25], highlights the importance of both laboratory and field testing. In tunnel tests, mosquitoes have prolonged contact with the net, while in hut trials, free-flying mosquitoes may have shorter, intermittent contact periods. This behavioral response could be due to the irritant or repellent effects of the insecticides, as observed in previous studies [26,27].

Insecticide resistance profiling revealed moderate to high intensity resistance to pyrethroids in both *An. gambiae s.l.* and *An. funestus* populations. The partial restoration of susceptibility following PBO pre-exposure indicates the involvement of metabolic resistance mechanisms, particularly oxidases. However, the incomplete restoration suggests the presence of additional resistance mechanisms, such as target-site mutations or other metabolic pathways. [5,28]. This complexity of resistance [29] emphasizes the value of combination nets deploying multiple active ingredients.

The differential retention of active ingredients has important implications for long-term efficacy: Alpha-cypermethrin retention (79.3%) exceeds WHO criteria and aligns with previous durability studies [30], PBO retention (74.1%) maintains sufficient synergist activity [1] and Pyriproxyfen retention (56.8%) may limit long-term sterilizing effects [15]). The multi-modal action of MiraNet® Combi provides both immediate (mortality and feeding inhibition) and long-term (sterilizing) effects. However, the lower retention of pyriproxyfen suggests potential trade-offs in long-term population effects. Recent modeling studies [13] indicate that even partial sterilizing effects can contribute to population reduction.

The combination of these compounds addresses multiple biological targets in the mosquito, potentially delaying the development of resistance to individual components [31]. The non-inferiority of MiraNet® Combi to established combination LLINs (Royal Guard® and Olyset™ Plus) in terms of mortality and blood-feeding inhibition is an important outcome. This indicates that MiraNet® Combi could be a valuable addition to the vector control toolbox, offering comparable efficacy to existing products while potentially providing operational or cost advantages [10]. The high blood-feeding inhibition (90–98%) observed across all LLINs, including after 20 washes, is a crucial finding. This suggests that even in areas with high pyrethroid resistance, ITNs continue to provide significant personal protection by preventing mosquito bites [26]. The additional sterilizing effect of pyriproxyfen in MiraNet® Combi and Royal Guard® offers a supplementary mode of action for population control, which could be particularly valuable in high-transmission settings [9,15,32].

This study has several strengths: comprehensive chemical analysis paired with bioassays, robust statistical design meeting WHO guidelines, field-relevant testing against wild pyrethroid-resistant vectors and direct comparison with currently recommended combination nets. However, important limitations should be considered: the nine-week trial duration may not capture seasonal variations, the focus on *An. funestus* limits generalizability to other vector species, small sample sizes for reproductive effects assessment and a lack of molecular resistance mechanism characterization.

Future studies should consider extended trial periods and multiple vector species to provide a more comprehensive assessment of LLIN performance [33]. Additional research priorities emerging from this work include the need for impact assessment against multiple vector species, cost-effectiveness studies compared to standard nets, investigation of resistance development risks and optimization of washing resistance, particularly for pyriproxyfen.

While triple-active ingredient LLINs offer improved control of resistant malaria vectors, their widespread deployment warrants continued evaluation of potential effects on non-target insects and the broader ecological environment. Ongoing resistance monitoring and post-marketing surveillance will be essential to ensure sustainable use.

## Conclusion

MiraNet® Combi demonstrated non-inferiority to both Royal Guard® and Olyset® Plus against pyrethroid-resistant *An. funestus*. The ITN showed superior mortality compared to standard MiraNet® while maintaining comparable blood-feeding inhibition. Chemical analysis revealed differential retention of active ingredients after washing, with alpha-cypermethrin and PBO showing higher retention than pyriproxyfen, though all components except pyriproxyfen exceeded WHO retention criteria. These findings support three key conclusions. First, MiraNet® Combi provides equivalent or better protection compared to existing combination nets despite high pyrethroid resistance. Second, the addition of PBO and pyriproxyfen to alpha-cypermethrin improves efficacy over standard pyrethroid-only nets. Third, while wash resistance of pyriproxyfen needs improvement, the overall performance meets WHO criteria for public health value. Future deployment should consider local resistance mechanisms and washing practices, while continued monitoring will be essential to track long-term durability and effectiveness. MiraNet® Combi represents a valuable addition to the vector control toolbox, particularly in areas where metabolic resistance mechanisms compromise standard pyrethroid-only nets.

## Abbreviations

EHTs: Experimental hut trials
GLP: Good laboratory practices
KEMRI-CGHR: Kenya Medical Research Institute_ Centre for Global Health Research
ITNs: Insecticide-Treated Nets
MN: MiraNet®
MNC: MiraNet® Combi
OP: Olyset® Plus
PBO: Piperonyl butoxide
PPF: Pyriproxyfen
PQT: Pre-Qualification Team
UN: untreated net
RG: Royal Guard
WHO: World Health Organization
